# POSITIVE ASPECTS OF CAREGIVING IN DIFFERENT CAREGIVER GROUPS

**DOI:** 10.1093/geroni/igac059.264

**Published:** 2022-12-20

**Authors:** Mengyao Hu, Dena Schulman-Green, Emma Zang, Bei Wu

**Affiliations:** New York University, New York, New York, United States; Rory Meyers College of Nursing, New York University, New York City, New York, United States; Yale Sociology Department, Yale University, New Haven, Connecticut, United States; New York University, New York, New York, United States

## Abstract

Previous studies have disproportionately focused on caregivers’ negative experiences while overlooking the positive aspects of caregiving (e.g., quality of caregiver – care recipient relationship, meaningfulness of caregiving, and family cohesiveness) especially for caregivers of older adults with cognitive impairment. Therefore, we aim to identify how positive aspects of caregiving varied by care recipients’ cognitive status (e.g., normal, mild cognitive impairment, dementia) and caregivers’ relation to care recipients (e.g., spouse, adult child, other family member). We applied multilevel mixed-effects models on pooled three-wave data from the National Study of Caregiving and the National Health and Aging Trends Study (N = 2,717). The findings suggested that dementia and spouse caregivers had worse relationship with their counterparts. Overall, future research needs to study caregiver’s experience integratively and focuses on caregiver’s individual need. Policy makers need to fulfill caregiver’s demands by establishing socially supportive programs.

